# The effect of combined training (theoretical-practical) of palliative care on perceived self-efficacy of nursing students

**DOI:** 10.1371/journal.pone.0302938

**Published:** 2024-07-11

**Authors:** Naiire Salmani, Fatemeh Keshmiri, Imaneh Bagheri

**Affiliations:** 1 Research Center for Nursing and Midwifery Care, Non-Communicable Diseases Institute, Nursing Faculty, Meybod Nursing School, Shahid Sadoughi University of Medical Sciences, Yazd, Iran; 2 Educational Development Center, Medical Education Department, Shahid Sadoughi University of Medical Sciences, Yazd, Iran; 3 School of Public Health, Shahid Sadoughi University of Medical Sciences, Yazd, Iran; 4 Research Center for Nursing and Midwifery Care, Department of Nursing, Non-Communicable Diseases Institute, Nursing Faculty, School of Nursing and Midwifery, Shahid Sadoughi University of Medical Sciences, Yazd, Iran; University of Eastern Finland: Ita-Suomen yliopisto, FINLAND

## Abstract

**Background:**

Nurses and researchers emphasize the importance of adding educational content of palliative care to nursing curricula in Iran as a means to improve the quality of care at the end of life and self-efficacy is considered as an important determinant in palliative care nursing. However, undergraduate nursing students are not sufficiently trained to achieve the qualifications required in palliative care. The aim of this study was to determine the effect of combined training (theoretical-practical) of palliative care on the perceived self-efficacy of nursing students.

**Methods:**

This is a semi-experimental study with a pretest-posttest design. Sampling was nonrandomized with convenience method and included 23 seventh-semester students. The intervention consisted of palliative care training for ten theoretical sessions and three practical sessions. Data were collected using demographic and the perceived self-efficacy questionnaires completed before and after the intervention. Data were then analyzed in the statistical SPSS 23 software using descriptive and analytical statistics.

**Results:**

The mean age of the samples was 22.78 (SD1.17). Most of the participants were male (56.5%) and single(91.3%). The findings showed that, perceived self-efficacy, psycho-social support and symptom management improved significantly after the intervention (p<0.05).

**Conclusion:**

Palliative care training can increase the nursing students perceived self-efficacy. Since nursing students are the future nurses of the care system, therefore, managers and planners can take a step towards improving the quality of nursing care by using palliative care training programs. Since nursing students will be future nurses in health care system, therefore, managers and planners can take steps to improve the quality of nursing care by using palliative care education programs.

## Background

According to the World Health Organization, “palliative care is an approach that improves the quality of life of the patients and their families facing problems associated with life-threatening illness through preventing and relieving the patient’s suffering and by identifying early identification, evaluation and treatment of pain and other physical, psychological and spiritual problems” [1].

This approach can be beneficial for different groups of patients, including patients with cancer, cardiovascular, lung diseases, acquired immunodeficiency syndrome, diabetes, kidney failure, neurological diseases and dementia [1]. In other words, palliative care can be applicable for all patients with dangerous diseases, regardless of age and type of disease, and regardless of the background [2].

The evidence shows that if the palliative care approach is implemented on time, it leads to the improvement of the quality of life, better symptom management [3–5], caregivers’ burden reduction, fewer hospitalizations, continuity and coordination of care, cost-effectiveness of care, and a good and peaceful death in the desired place of the patient [6–8]. However, providing palliative care requires training [9]. However, providing palliative care requires training [9] and increased professional training and improved information exchange are key determinants that facilitate the integration of palliative care into the health care system [10].

Therefore, based on an international consensus, the integration of palliative care education in the teaching program of nursing curricula in undergraduate and graduate courses has become mandatory [11]. Despite this broad consensus, it is still a complex issue for beginning education in palliative care at the undergraduate level [12, 13]. In Iran, palliative care is still a major challenge for the health system [14] and is not performed in an integrated way for patients [15].

Despite the importance of the topic and the recommendations of experts in this field, no coherent actions have been taken in the field of palliative care education. Also, concepts such as death, care and family have been emphasized in the nursing undergraduate curriculum [16]. Meanwhile, studies conducted in the field of palliative care show that Iranian nurses have a neutral or negative attitude towards palliative care [17] and they have relatively poor knowledge [18].

So early integration of palliative care education impacts upon students perceived preparedness for practice and positively influences their attitudes to palliative care provision [17].

Moreover, a challenge raised in this field is the lack of appropriate educational models and operational plans [19]. Even though a century has passed since the history of nursing education in Iran, the situation of nurses’ education in the field of palliative care remains unfavorable [16].

Furthermore, inadequate training of nursing students in this regard results in them being unprepared for their future roles as palliative care providers upon graduation [20]. This is despite the fact that providing students with appropriate scientific services and training can lead to a sense of self-confidence and self-belief in doing assigned tasks, which is called self-efficacy [21]. In nursing profession, self-efficacy in the educational system evaluates the success of the training; and the level of self-efficacy can be used to measure the ability of nursing students during their studies to become health workers, that is, professional nurses [22]. Also, self-efficacy is considered as an important determinant in palliative care nursing [23]. However, undergraduate nursing students are not sufficiently trained to achieve the qualifications required in palliative care [24].

The level of self-efficacy of nursing students is related to their stress level and how they overcome difficult situations; so that the low self-efficacy of the student leads to experiencing too much stress [25], avoiding behaviors in dealing with the patient in need of palliative care [26], having low self-confidence and avoiding asking for help in case the patient’s condition worsens [24].

Educational institutions are expected to use different methods of active learning in palliative care, such as watching videos focused on the process of communication between nurses and patients and families, practicing listening to the patient’s symptoms, simulating a case to learn how to correctly assess the patient, Use nursing diagnosis and interventions [27].

Therefore, focusing on the importance of palliative care education and the lack of palliative care education in Iran’s undergraduate nursing curriculum, the present study aimed to determine the effect of a combined (theoretical/practical) palliative care educational program on the self-efficacy of nursing students.

## Methods

### Study design

This study was before-after design and carried out by non-probability available sampling.

### Study setting, sampling, intervention

This study was carried out at Meybod Nursing School, Yazd, Iran. 23 nursing student of the 7th semester were selected using a census sampling.The inclusion criteria included willingness to participate in the study and passing all theoretical units and internships of specialized nursing courses. 27 students met the criteria for entering the study; 4 of them were excluded from the study due to receiving approval to transfer to another faculty and not being able to attend the training sessions. Exclusion criteria included absence of more than one session in theory sessions or practical sessions. So 23 participants entered the study. This study was conducted after receiving the ethic cod of IR.NASRME.REC.1400.397 from the National Center for Strategic Research in Medical Education. In order to conduct the study, the participants were first informed about the objectives and the method of conducting the study. After receiving verbal consent and completing the written consent form, the participants were provided with study questionnaire. The confidentiality of the collected information was emphasized and the participants were informed that they could withdraw from the study at any time.

The design of the palliative care training program was compiled by the research team based on the search for palliative care training programs implemented in other universities, the review of articles related to the palliative care training of nursing students published and available. In the next stage, the research team held a meeting with the Vice-Chancellor and the director of the faculty and made the necessary arrangements for the implementation of the training program. Considering that at the beginning of the 7th academic semester, students must complete 8 units under the title of nursing internship units for adults and the elderly, and the internship in the oncology department was a part of the education planning based on the curriculum, so at the time of the study, it is possible for students to attend the oncology department.

The studied samples participated in an orientation session at the beginning of the semester (September to November 2022). In this session, the students were provided with the necessary information regarding the way of conducting the palliative care training course (goals, focus of training, method of training).

According to the prepared educational program, lecturers with expertise in palliative care were invited to hold theoretical training sessions. The theoretical sessions, which were held in four weeks, included ten sessions and each session lasted for two hours. The first lecturer (Doctorate in nursing, with a doctoral thesis in the field of palliative care) held two theoretical sessions- each session lasted for two hours- related to the concept of palliative care, the principles of providing palliative care and communication with the patient.

The second instructor (Palliative Care Fellowship) taught six two-hour sessions related to symptom management (physical-psychological-social-spiritual). And the third lecturer (master’s degree in psychiatric nursing) taught two sessions- each session lasted for two hours- related to the concept of death, bereavement and other related concepts in this field. All lecturers used the method of presenting the film, presenting the case (case report), discussion and conversation along with the lecture.

After the theoretical sessions, the practical program was designed. In nursing education programs, theory and practice are integrated conducted. Students get the necessary theoretical information at school; however, they also try to improve their clinical judgment ability by transferring the theoretical knowledge to practice to transform the information to the behavior and learn the whats, whys, and hows [28]. Experiences, expectations, and recommendations of the students about theoretical and practical education can contribute to the more effective nursing educational processes [29].

The practical program based on Bandura’s theoretical framework for self-efficacy resources. Bandura (1977) identified four main sources of self-efficacy: performance outcomes, vicarious experience, verbal persuasion, and emotional state [30].

In order to achieve these four sources, the practical part of the training was designed in such a way that students were divided into six groups [3–4 students in each group]. Each group was subjected to practical training for three days (from 8:00 AM to 12:00 PM).

This part of the intervention was carried out in the oncology department of two selected hospitals affiliated to the university with senior nurses (The trainers selected for cooperation was nurses with at least ten years of work experience in the oncology department with a master’s degree in medical—surgery, who had completed palliative care workshops held in the hospital).

To provide a resource for " performance outcomes," senior nurses identified palliative care situations and they specified the activity program of the students in order to provide opportunities to practice based on the knowledge they have learned and develop beliefs and opinions about their abilities to provide palliative care.

In this regard, under the supervision of the supervisors, each student communicated with a certain patient (Patients with various types of cancer, including leukemia, lymphoma, stomach, colon, liver, and breast) and to identify the symptoms, the patient was evaluated. Then symptom management was done. The second source was " vicarious experience ". To provide this source, students were guided to observe the performance of a supervisor providing palliative care in order to learn the appropriate methods of providing palliative care as a model, and identify similarities between themselves and observed characteristics, and believe in their abilities.

The third source was "verbal persuasion”. To provide this source, the supervisor was present when the student encountered the patient and provided care, and by giving appropriate feedback regarding the student’s ability, the supervisor tried to encourage the student to continue trying to improve the learned skills.

The fourth source was "emotional state”. By evaluating the student’s emotional state in terms of stress, anxiety, fear and worries while facing the patient and providing palliative care, the supervisor tried to identify the psychological pressures and helped to improve the psychological and emotional condition of the students with appropriate intervention including: discussing with students about the causes of psychological stress, encouraging students to support their peers in providing care and providing an opportunity to vent their emotions from interacting with patients. This training process was repeated during the three days of being at the bedside. During the process, the supervisor, by giving feedback to the students regarding the improvement in their performance to provide palliative care to patients, solved the weaknesses of the students; and, by confirming the strengths of the student’s performance and by providing positive verbal and non-verbal feedback, the supervisor encouraged the students’ capabilities.

### Data collection tool and data analysis

The demographic information form included age, gender and marital status. The self-efficacy measurement scale in palliative care was first designed by Phillips et al. (2011) and its validity and reliability were approved [31]. In Iran, this tool has been translated by Dehghani et al. and its validity and reliability have been checked and approved [32].

The tool has two dimensions. The first dimension measures psychosocial support with 6 items and the second dimension measures symptom management with 6 items. The reliability of the tool, using Cronbach’s alpha coefficient, is reported as 0.84 for the first dimension, 0.78 for the second dimension, and 0.70 for the whole tool. This scale is graded based on a 4-option Likert scale: feeling certain in performing care independently (4 points), doing it independently with minimal consultation (3 points), confidence in performing care with more supervision (2 points), the need to receive more training (1 point). The highest score is 48 and the lowest score is 12. A high score indicates better self-efficacy. In this study the reliability of the tool was checked and confirmed using Cronbach’s alpha coefficient (0.80 for the first dimension, 0.75 for the second dimension and 0.70 for the whole tool).Self-efficacy questionnaire was completed before and after the intervention. Before the start of the intervention, the questionnaires were given to the samples by the first author at the beginning of the theory session first in the classroom and completed. The second time(after), the questionnaires were completed after the completion of the theoretical and practical classes.

The collected data were analyzed by SPSS version 23 statistical software using descriptive and analytical statistical tests (Paired t-test) and a significance level of 0.05 were considered. Kolmogorov Smirnov test was used to check the normality of the data (p>0.05).

## Results

The mean age of the participants was 22.78 (SD1.17) years. 13 participants (56.5%) were males and 10 participants (43.5%) were females and the majority (91.3%) were single (Table 1).

**Table 1:** Demographic characteristics of the samples.

Variable		n	%
Gender	Man	13	56.5
	Woman	10	43.5
Marital Status	Single	21	91.3
	Married	2	8.7

The mean score on perceived self-efficacy after the intervention was 37.56 (SD1 9.29), which was higher than before the intervention 24.52 (SD 8.32). And a significant statistical difference was seen (P = 0.021).

The mean score on perceived self-efficacy in the field of symptom management after the intervention was 19.48 (SD 5.13) more than before the intervention 12.96 (SD 5.12). And a significant statistical difference was seen (P = 0.034).

The mean score on perceived self-efficacy in the field of psychosocial support after the intervention was 18.08 (SD 4.92) more than before the intervention 11.56 (SD 3.91)and a significant statistical difference was seen (P = 0.041) (Table 2).

**Table 2:** Comparison of perceived self-efficacy before and after the intervention.

Field	Before		after	
	Mean	SD	Mean	SD
psychosocial support	11.56	3.91	18.08	4.92
Symptom management	12.96	5.12	19.48	5.13
Total self-efficacy	24.52	8.32	37.56	9.29

## Discussion

The purpose of this study was to investigate the effect of combined training (theoretical-practical) of palliative care on self-efficacy of nursing students. The results showed that combined training (theoretical-practical)improves the nursing students’ perceived self-efficacy score.

In this study, the combined learning method (theoretical and practical) was used in different ways, including watching movies, discussing in small groups, case-based learning, bedside learning. In fact, the purpose of using these methods was that the nursing student would be able to acquire the necessary knowledge and skills to take care of patients in need of palliative care.

Meanwhile, important concepts such as general principles of palliative care [33], assessment and management of pain and other symptoms [34] and communication [33] were included in the educational program. Moreover, since trying to keep students active and creative in the teaching-learning process and achieving self-efficacy is one of the most important things in choosing a teaching method in universities, professors should pay special attention while choosing a teaching method and they should use certain methods to improve self-efficacy [35].

Therefore, in the present study, a combination of different educational methods was used. And in the practical part of education based on Bandura’s theory, an attempt was made to provide four sources that develop self-efficacy. So that at first, by holding theory workshops, students were provided with the opportunity to acquire knowledge related to palliative care.

Previous studies also acknowledged that it is necessary to acquire knowledge and receive training in relation to communication skills and symptom management, in order to gain self-efficacy in palliative care [36].

After the theoretical training, a situation was prepared so that during the practical training, the students would be present at the patient’s bedside so that under the supervision of the supervisor, through an interview with the patient, they detected the symptoms, planned and implemented the symptoms management in order to provide an opportunity to accumulate experiences.

Billings et al. also mentioned this issue in 2013 and introduced students’ clinical experiences as an important predictor of students’ self-efficacy [36]. On the other hand, according to Carper’s theory, nursing knowledge (ways of knowing) is improved through personal, experimental, ethical and aesthetic knowledge; and, when experimental knowledge is expanded through aesthetic and personal knowledge and ethical concerns are resolved through reflection and education, a better self-efficacy will be experienced to provide palliative care [37].

Vicarious experiences gained during training courses and clinical placements help nursing students gain confidence to handle similar situations and gaining positive experiences in the field of providing palliative care can increase the self-efficacy of nursing students [24]. The presence of a supervisor in the practical training of palliative care as a model provides a "vicarious source" and as Bandura states, if people see that others can do something, then they convince themselves that they can reach it and at least some improvement in their performance is created [38].

The next source was "verbal persuasion" of the students. Bradbury-Jones et al. state that the instructor’s support in clinical settings plays an essential role in developing student empowerment; and receiving positive verbal feedback can strengthen a person’s abilities to perform a specific action [39].

In the current study, the supervisor’s effort to encourage students while providing care was a step to provide this resource. The last source was emotional state Emotional arousal can also affect a person’s expectations of personal competence [40] strategies that provide emotional support are effective in enhancing self-efficacy. Conversely, stress, anxiety and negative experiences can reduce self-efficacy.

In the present study, the students were under the psycho-emotional support of the supervisor, and the supervisor provided the opportunity to express their feelings and use appropriate methods to release negative emotions. On the whole, the designed educational intervention was able to increase the self-efficacy of nursing students generally in both areas [symptom management, psycho-social dimension]. In line with this finding, Adriansen et al. and Carey et al. also state that providing training programs over a course, allows nurses to practice what they learn and then reflect on their experiences, ponder over their professional practice, encourage them to change their attitudes and actions, evaluate themselves and determine whether they have met the needs of their patients or not [41, 42]. After completing palliative care training programs, they report increased confidence in assessment and symptom management skills, as well as feeling empowered to approach doctors about their patients’ concerns [43].

Bassah et al also -during a study aimed at explaining students’ perception of the implementation of the palliative care educational program- stated that the use of interactive learning methods such as role playing, group discussion and case study is effective in increasing their learning in palliative care education [44]. Dehghani et al also reported an increase in self-efficacy in general and in both areas [symptom management, psycho-social dimension] by implementing a training program related to palliative care for nurses [32].

This study had strengths and weaknesses. The weakness of this study was no control group in this study, due to the existence of only one group of nursing students of the seventh semester who had passed all theoretical and internship units in Meybod School of Nursing and were ready to enter the nursing internship course. Not having a control group is one of the limitations of the present study. Therefore, it is recommended to include a control group in future studies to investigate the effect of the educational intervention in order to obtain more reliable findings. Also, in this study, there was no follow-up after the intervention. therefore, re-evaluating the effect of educational intervention on self-efficacy after a certain period of time is suggested in future studies to determine the durability of the intervention’s effects.

The strong point of the study was that the research project approved in the Strategic Research Center of Medical Education affiliated to the Ministry of Health and Medical Education of Iran and currently this center emphasizes the implementation of innovative research in the educational programs of students, therefore this study with the design of the Palliative care educational program made a change in the internship program of nursing students and the students became familiar with the concept of palliative care and how to provide it to cancer patients.

## Conclusion

The results showed that the implementation of an educational program can improve nursing students’ perceived self-efficacy in palliative care. because this study was in the time frame of the training course of the internship students, it was simply implemented and accepted by the students, which can be of interest to the planners and managers of the nursing education system. So by planning and holding consecutive training courses in the field of palliative care, nurses with high self-efficacy can be trained and prepared to provide service in the palliative care system.
