# Correction: The effect of combined training (theoretical-practical) of palliative care on perceived self-efficacy of nursing students

**DOI:** 10.1371/journal.pone.0308270

**Published:** 2024-07-30

**Authors:** Naiire Salmani, Fatemeh Keshmiri, Imaneh Bagheri

There are errors in the author affiliations. The correct affiliations are as follows:

Naiire Salmani^1,2^, Fatemeh Keshmiri^1,3,4^, Imane Bagheri^1,5^

**1** National Agency for Strategic Research in Medical Education, Tehran, Iran, **2** Meybod Nursing School, Shahid Sadoughi University of Medical Sciences, Yazd, Iran, **3** Medical Education Department, Educational Development Center, Shahid Sadoughi University of Medical Sciences, Yazd, Iran, **4** School of Public Health, Shahid Sadoughi University of Medical Sciences, Yazd, Iran, **5** Nursing Faculty, Department of Nursing, School of Nursing and Midwifery, Shahid Sadoughi University of Medical Sciences, Yazd, Iran.

The Data Availability statement for this paper is incorrect. The correct statement is: All relevant data are available from the Figshare repository at https://doi.org/10.6084/m9.figshare.24993546.

The funding statement for this paper is incorrect. The correct funding statement is as follows: The project was funded by the National Agency Strategic Research in Medical Education. Tehran. Iran (Grant No. 4000316). The grant supported the data collection process. The funders had no role in the design of the study and collection, analysis, interpretation of data, or preparation of the manuscript. The report of the study’s findings is sent by the author to the funder at the end of the study.
